# The Global Threat from the Irreversible Accumulation of Trifluoroacetic Acid (TFA)

**DOI:** 10.1021/acs.est.4c06189

**Published:** 2024-10-30

**Authors:** Hans Peter H. Arp, Andrea Gredelj, Juliane Glüge, Martin Scheringer, Ian T. Cousins

**Affiliations:** † Norwegian Geotechnical Institute (NGI), 0484, Oslo, Norway; ‡ Department of Chemistry, Norwegian University of Science and Technology (NTNU), 7491, Trondheim, Norway; # Institute of Biogeochemistry and Pollutant Dynamics, ETH Zürich, 8092 Zürich, Switzerland; ∥ RECETOX, Masaryk University, 625 00 Brno, Czech Republic; ⊥ Department of Environmental Science, Stockholm University, SE-10691 Stockholm, Sweden

**Keywords:** trifluoroacetic acid, multigenerational exposure, PFAS, PMT, vPvM, environmental monitoring

## Abstract

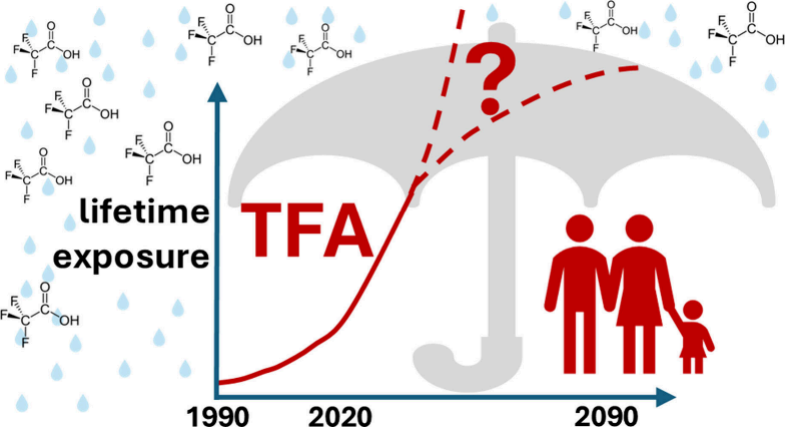

Trifluoroacetic acid (TFA) is a persistent and mobile substance that has been increasing in concentration within diverse environmental media, including rain, soils, human serum, plants, plant-based foods, and drinking water. Currently, TFA concentrations are orders of magnitude higher than those of other per- and polyfluoroalkyl substances (PFAS). This accumulation is due to many PFAS having TFA as a transformation product, including several fluorinated gases (F-gases), pesticides, pharmaceuticals, and industrial chemicals, in addition to direct release of industrially produced TFA. Due to TFA’s extreme persistence and ongoing emissions, concentrations are increasing irreversibly. What remains less clear are the thresholds where irreversible effects on local or global scales occur. There are indications from mammalian toxicity studies that TFA is toxic to reproduction and that it exhibits liver toxicity. Ecotoxicity data are scarce, with most data being for aquatic systems; fewer data are available for terrestrial plants, where TFA bioaccumulates most readily. Collectively, these trends imply that TFA meets the criteria of a planetary boundary threat for novel entities because of increasing planetary-scale exposure, where potential irreversible disruptive impacts on vital earth system processes could occur. The rational response to this is to instigate binding actions to reduce the emissions of TFA and its many precursors.

## Abstract 2

Rapidly increasing TFA concentrations, coupled with TFA’s extreme persistence, mobility and the possibility of irreversible impacts, should prompt action to reduce emissions of TFA and its precursors.

## Introduction

Trifluoroacetic acid (TFA) belongs to the subclass of per- and polyfluoroalkyl substances (PFAS) known as ultrashort-chain perfluoroalkyl acids (PFAAs). TFA is by far the most abundant PFAS in the environment.^1−6^ Neuwald et al. demonstrated that TFA accounted for more than 90% of the total PFAS mass (of 46 individual PFAS analyzed) in various drinking water sources in Germany.^2^ Tian et al. observed that concentrations of TFA and perfluoropropionic acid (PFPrA) in air, dry-deposition particles, and plant leaves surrounding two landfills in China were an order of magnitude higher than those of the 21 other PFAS analyzed.^4^ Chen et al. measured 25 PFAS around two fluorochemical manufacturing plants in China, covering 8 different media (air, various water, soil, dust, plant leaves, sediment), with TFA concentrations being consistently 1–2 orders of magnitude higher than those of other PFAS.^5^

An initial wave of scientific interest in the environmental fate and effects of TFA started around the mid-1990s, due to novel fluorinated refrigerants (hydrofluorocarbons (HFCs) and hydrochlorofluorocarbons (HCFCs)) being introduced to the market after the ozone-depleting chlorofluorocarbons (CFCs) were phased out under the 1987 *Montreal Protocol on Substances that Deplete the Ozone Layer*.^7−12^ Many of the fluorinated refrigerants are referred to as F-gases (encompassing gases with an R–CF_3_ moiety, R–CF_2_–R moiety, or inorganic fluorides). When F-gases or other fluorinated organic substances contain a C–CF_3_ moiety that is resistant to biochemical or photochemical degradation, TFA will commonly be a terminal degradation product. In recent years, interest in TFA has been re-established due to rapidly increasing concentrations observed in remote locations, as well as its ubiquity in drinking water sources and human blood.^2,13−15^

Since the 1990s, it has been suggested that hazard-related concerns of TFA and other short-chain PFAAs are much lower than those of PFAAs with longer perfluoroalkyl chains, which are more bioaccumulative and generally more toxic.^8,9,16−23^ However, these early reports did not consider TFA’s ubiquitous accumulation in the environment, in particular its observed accumulation in water resources and bioaccumulation in various plants, including crops. Although there are fewer toxicological data compared to long-chain PFAAs, we maintain that there are more than sufficient data to conclude that TFA poses a risk to humans and the environment and meets the criteria of a planetary boundary threat for novel entities.^24−27^ We will present evidence for this based on TFA’s (1) increasing planetary exposure, which is (2) irreversible and increasing due to emissions from many sources and could thereby cause (3) long-lasting disruptive effects on human health and vital earth system processes. Our analysis leads to the conclusion that policy, industry initiatives, and innovation actions should be enacted globally to reduce TFA emissions as soon as possible to protect future generations from potential irreversible effects of TFA accumulation.

## Increasing Planetary Exposure

A review of the scientific literature was conducted to obtain an overview of average and maximum concentrations in diverse environmental media (see the Supporting Information (SI) for a full methodology and collected data). In brief, 43 studies reporting on TFA concentrations spanning from the late 1990s to the 2020s were selected, and monitoring data were analyzed. Collectively, these data indicate that TFA exposure is widespread and is increasing.

Recent median concentrations of TFA in precipitation were measured at 0.29 μg/L in the USA,^28^ 0.21 μg/L in Germany,^29^ and 0.70 μg/L in Fuxin, China.^5^ The median drinking water concentrations reported in the recent peer-reviewed literature vary from 0.08 μg/L in the USA (Indiana)^18^ to 1.5 μg/L in Germany,^2^ and 0.23 μg/L in samples from 19 different countries other than Germany.^30^ This has relevance to TFA being included within regulatory definitions of “total PFAS”. These median concentrations are either similar to or higher than the proposed limits of total PFAS in drinking water in the EU draft recast Drinking Water Directive,^31^ which places a threshold of 0.5 μg/L for total PFAS.^32^ A recent report measuring TFA in European surface water and groundwater recorded an exceedance of the 0.5 μg/L threshold in 79% of the samples, with more than 98% of the detected mass of total PFAS being TFA.^32^ A Swiss study reported detecting TFA in 560 out of 564 drinking water samples in 2023, with 69% of the concentrations above 0.5 μg/L, among which were 2 samples with TFA concentrations above 9 μg/L and 75 between 1 and 9 μg/L.^33^

If there is TFA contamination in soil or water, short and ultrashort PFAAs undergo rapid uptake and bioaccumulation in crops and other plants, particularly in their aerial compartments.^34−37^ Chen et al.^5^ detected concentrations of up to 3800 mg/kg_dw_ of TFA in plants in the vicinity of a fluorochemical industrial site in China with an average field bioaccumulation factor of 13,000. In general, TFA has been enriching in conifer needles,^5,38^ maize,^5,39^ leaves of various tree species,^4,5^ and some wetland species.^40^ Consequently, high concentrations of TFA in plant-based foods^41^ and plant-based beverages such as juice,^42^ beer, and tea^30^ were reported, indicating that the ingestion of plant-based foods and beverages could be a substantial route for human (and animal) exposure.

TFA was detected in human blood from China^43^ with median concentrations of 8.46 μg/L, similar to the levels of long-chain PFAAs, although TFA is not considered bioaccumulative according to regulatory criteria (which typically refer to bioaccumulation in aquatic species entering the food chain and not to bioaccumulation in plants).^44^ A similar study in the USA reported a median of 6 μg/L and a maximum of 77 μg/L,^18^ where TFA alone had a 57% contribution to the total mass of 39 detected PFAS in human serum. Thus, the concentrations of TFA in nonoccupationally exposed US citizens are similar to the concentrations of bioaccumulative legacy long-chain PFAAs (e.g., PFOS, PFHxS, PFNA, PFDA) measured in the serum of occupationally exposed workers.^45^ Because of high uptake, TFA reaches levels in human serum that are much higher than its low bioaccumulation potential in aquatic species would indicate.

**Figure 1:**
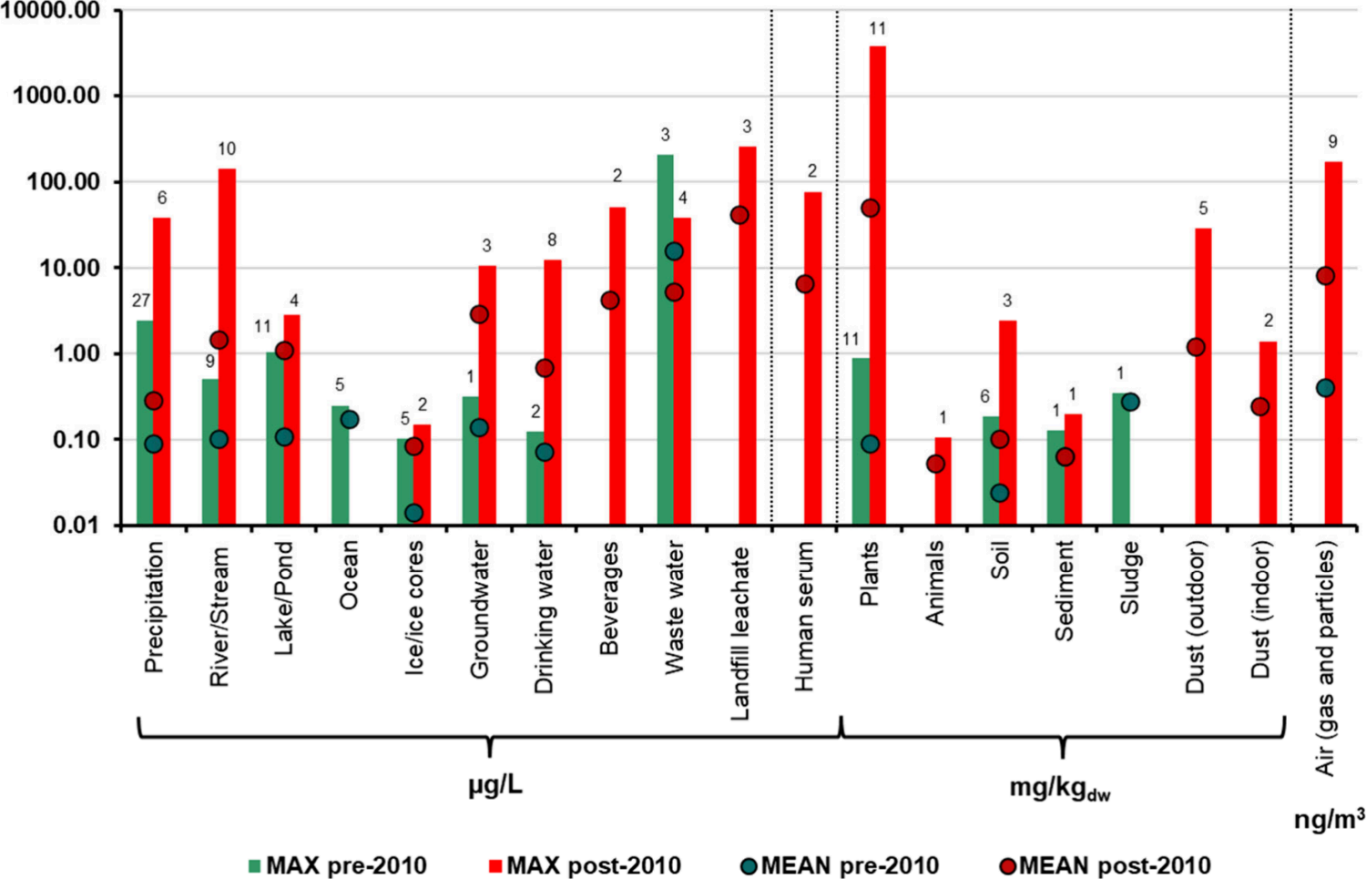
Comparison of TFA concentrations detected in different media summarized as before 2010 (green) and after 2010 (in red). Reported values of maximum concentrations found in the literature review are shown as vertical bars and mean values of reported monitoring means and medians are shown with overlapping dots. Numbers indicating the number of summarized individual data points are shown above the corresponding bars. In cases of either green or red bars and dots missing, data were not available for the given media and/or time frame. For air concentrations before 2010 only two studies were available, one of which reported only mean measured concentrations (see Table S1 for the full data set and Figure S3 for air data presented as concentrations from individual studies).

To demonstrate the change in concentrations and trends over the years, Figure 1 presents a comparison of pre-2010 and post-2010 data (by the reported year of sampling, not the year of publication). This cutoff year of 2010 was chosen because there was a slump of interest in TFA in the peer-reviewed literature between the years 2008 to 2012. Prior to this time, research on TFA attracted attention in relation to the introduction of F-gases around the late 1990s to replace CFCs. In more recent years, TFA has attracted increased attention due to it more commonly being included and detected during PFAS monitoring campaigns. The first of January 2010 also coincides with the final deadline for stopping the last remaining production of CFCs;^46^ even though the transition to F-gases occurred much before this time.^15^

When pre- and post-2010 concentration data are available in a specific medium (Figure 1), an increase in TFA by over an order of magnitude in both maximum and mean concentrations in several environmental compartments is evident, including for precipitation, rivers and streams, groundwater, drinking water, soil, and plants. While atmospheric media were the main focus of monitoring studies dated pre-2010 (especially precipitation), more data from other media became available after this cutoff year—with new media relevant to human exposure (e.g., plant-based beverages, crops, indoor and outdoor dust, human serum), ecosystem exposure (e.g., animals such as locusts, autochthonous tree species), as well as other media that were rarely measured before (e.g., drinking water). Comprehensive figures with concentrations across different media are available in the SI (Figures S1–S4).

More detailed time trends are available at specific locations. Urban surface waters around Beijing were sampled in 2002^47^ and were resampled in 2012,^48^ showing up to a 17-fold increase in TFA concentrations over 10 years, while tap water detections went from nondetection to 0.16 μg/L in 2012.^48^ Freeling et al. reported an increase in wet deposition in Germany from 22 to 30 t/year during 1995–1996 to 68 to 98 t/year in 2019.^29^ Pickard et al. used dated Arctic ice cores to show a rapid increase in deposited TFA after the entry-into-force of the Montreal Protocol, from nondetection or a few ng/L in segments before 1989 to 0.13 and 0.15 μg/L in segments dated 2015 and 2017, respectively.^15^ In 2021, Cahill et al. reported a 6-fold increase in TFA in a stream transect in California since 1998.^49^ Freeling et al. analyzed archived leaf samples of different tree species for TFA and observed increases by factors of up to 12.5 in TFA concentrations in some species from 1989 to 2020.^50^ A study in indoor and outdoor dust found a 4-fold increase in TFA between 2013 and 2017.^51^

## An Irreversible Burden from Multiple Sources of Emissions

Based on its persistence and high mobility due to lack of sorption,^13,52^ TFA’s ultimate recipient is the Earth’s hydrosphere. As TFA is omnipresent in all water bodies, from groundwater, oceanic water, ice cores, drinking water to bottled water,^1,2,15,30,48,53−55^ dilution in many ways has already occurred, with the only substantial remaining dilution medium being the deep ocean. Similarly, enrichment in plants will follow constantly increasing TFA concentrations as water and soil concentrations increase. With limited processes for elimination from plant tissues because of TFA’s persistence (no biotransformation) and its lack of volatility (as it is a strong acid in water and fully ionized), constant passive uptake from soil pore water via the transpiration stream can be expected for the lifetime of the plant as long as there is an input of TFA available. This theory is supported by findings for other short- and ultrashort-chain PFAAs^36^ and is in agreement with the results by Zhang et al.,^56^ who did not observe a steady-state concentration being reached for TFA in their wheat experiments.

Exposure of TFA within agricultural and other ecosystems has not been assessed to the same extent as it has for other, longer-chain PFAAs;^57−62^ however, it is inevitable that with increasing emissions on the global scale, the mean TFA concentrations in humans (serum), animals and plants will increase. The only potential environmentally relevant pathways of TFA degradation are overtone photodissociation in the troposphere and biologically catalyzed conversion processes that convert TFA to fluoroform (CF_3_H).^63^ This is likely negligible in terms of TFA mass loss, as TFA is readily scavenged from the atmosphere by wet (and to a lower extent, dry) deposition, before it spreads rapidly in the terrestrial hydrosphere.^12,48,64^ Nevertheless, considering the high TFA concentrations, even a low conversion of TFA to fluoroform may have an observable impact on atmospheric fluoroform concentrations.^63^ Methods to remove TFA from water are expensive and often inefficient due to TFA’s persistence and mobility.^53,65−67^ In some cases, water purification methods as common as ozonation and chlorination are also a source of TFA, depending on the presence of TFA-precursors.^53^ The most effective solution would be reverse osmosis (RO) to upconcentrate TFA, followed by some intensive destruction techniques. However, RO treatment of water, particularly wastewater, compared to other forms of water treatment is expensive,^68,69^ requires a substantial amount of energy^70^ and typically results in 50% water loss.^71^ The necessity of RO for water treatment is also in contradiction to Article 7(3) of the EU’s Water Framework Directive, which states “Member States shall ensure the necessary protection for the bodies of water identified with the aim of avoiding deterioration in their quality in order to reduce the level of purification treatment required in the production of drinking water”.^72^ Destructive techniques for concentrated TFA, such as incinerating RO concentrates or even biomass containing TFA, may lead to harmful byproducts, including fluoroform (a potent greenhouse gas with a GWP of 14,800 times that of CO_2_ over 100 years).^63,73^

We mentioned above that one source of increasing TFA concentrations is the increased use of F-gases used as refrigerant chemicals; it is worth looking into this in more detail. Refrigerant chemicals have a history of being problematic. CFCs were originally introduced as “safer”, alternative refrigerants to ammonia, sulfur dioxide and methyl chloride, until it was discovered that CFCs were volatile enough to reach the stratosphere and release Cl radicals that lead to the decomposition of stratospheric ozone, particularly over Antarctica.^74−76^ In this manner, CFCs were a planetary boundary threat associated with severe impacts.^77^ To protect the ozone layer from poorly reversible depletion, the Montreal Protocol^78^ required CFCs used in refrigeration and air conditioning systems to be substituted with HCFCs and HFCs^79^ or natural refrigerants. HCFCs have lower ozone depletion potential than CFCs, and HFCs have no ozone depletion potential; however, HFCs have a high global warming potential (GWP).^79^ Further amendments to the Montreal Protocol and the European F-gas Regulation aimed to replace the high-GWP F-gases with low-GWP alternatives.^80^ Nevertheless, the substances proposed as replacements–hydrofluoroolefins (HFOs) and even currently applied HFCs–add to the problem because many HFOs and HFCs can be either partially (e.g., HFC-134a, HFC-143a, HFC-1234ze, HCFO-1233zd) or completely (e.g., HFC-227ea, HFO-1225ye(E), HFO-1225ye(Z), u-HCFC-1224yd(Z), and HFO-1234yf) transformed to TFA.^81,82^ The use of HFO-1234yf was estimated to be responsible for 6900 t/year emissions of TFA in 2020 in the EU alone, with a potential increase in emissions up to 47,650 t/year by 2050.^81^ This shift in F-gases could cause emissions of TFA to increase by orders of magnitude in the coming years,^81^ along with a corresponding contribution to climate impacts due to the formation of more fluoroform from TFA in the troposphere.^63^

Another major source of TFA is direct emissions from industrial production and processes. For example, TFA is registered under EU’s Regulation 1907/2006 on the Registration, Evaluation, Authorisation and Restriction of Chemicals (REACH) as manufactured and/or imported into the EU in volumes ranging from 100 to 1000 tons per year.^52^ Additionally, TFA is used as an intermediate, but this usage does not require the registration of production volumes. Xie et al. measured TFA in the surrounding environment (surface water, groundwater, air, dust, soil) of three fluorochemical plants near Jinan, China, that manufacture inorganic fluorine products, TFA and fluoride-containing intermediates.^83^ Whereas Xie et al. reported surface water concentrations up to a maximum of 2.6 μg/L,^83^ concentrations up to 7500 μg/L were recently reported in surface water of the river Arias in the vicinity of a production plant producing TFA and related PFAS in Southern France.^84,85^

In addition to F-gases and direct TFA production,^83−85^ there are several other precursors contributing to the accumulation of TFA in the environment, including a variety of groups of chemicals containing the C–CF_3_ moiety, such as agrochemicals,^53,86−88^ pharmaceuticals,^53^ (fluoro)polymers,^89−91^ and other PFAS.^13,53,92^ TFA and its precursors can be emitted from point sources such as the (fluoro)chemical industry,^5,53^ wastewater treatment plants, landfills,^4,93,94^ and sites with a history of the use of aqueous film-forming foams (AFFFs).^93,95^ The German Environment Agency (Umweltbundesamt, UBA) published estimates for TFA emissions from sources in Germany.^96^ The available data indicate that refrigerants and blowing agents were the largest quantifiable source of TFA with emissions at ∼2000 t/year in Germany, followed by pesticides at ∼457 t/year (mainly flufenacet, diflufenican, and fluazinam), and human pharmaceuticals at ∼29 t/year. Emissions from other sources, such as direct production of TFA, biocides, veterinary pharmaceuticals, and fluorochemicals in products could not be quantified in that study.^96^ There have been speculations about natural sources of TFA, but these have been discredited by Joudan et al.,^97^ who demonstrated that only increasing anthropogenic sources of TFA can explain its exponential increase in recent decades. A source of TFA that has been recognized since the 1990s and is garnering increasing attention is the transformation products from the thermal destruction of fluoropolymers,^98^ as well as treatment of PFAS by destruction methods.^53,99,100^ TFA formation yields of diverse PFAS destruction methods are often unknown and this should therefore be investigated when developing new destruction methods for PFAS.^16^ TFA has been dubbed a “Substance from Multiple Sources” by Nödler and Scheurer,^101^ highlighting the difficulty in ascertaining the main sources of TFA in monitoring studies of water resources.^14,19^

## Disruptive Effects on Human Health and Earth System Processes

There may be disruptive effects of TFA on human health or on Earth system processes that are currently unknown, similar to how the effects of longer-chain PFAAs on human health and CFCs on the ozone layer were initially unknown by the broader scientific and regulatory community. Overcoming this ignorance is the hardest part of understanding that a certain novel entity that is irreversibly increasing in concentrations globally may pose a planetary boundary threat.^25^ Such effects cannot be fully described or anticipated by established paradigms of hazard and risk assessment. However, as those paradigms are commonly used in defining regulatory human and ecosystem health thresholds for chemicals, this is where we start our analysis of TFA potentially causing a disruptive impact on a planetary scale.

### Known Health and Environmental Thresholds

A recent review by Dekant and Dekant^17^ summarized the mammalian toxicity of TFA, referring specifically to human toxicity. Most toxicity tests on which human health risk assessments were based are rat studies, which found mild liver hypertrophy (increased size of the liver) as the lead effect at daily doses of > 1000 mg/kg_bw_ in a 90-day feeding study. More human-relevant tests directed toward a mechanistic understanding and not only toward filling regulatory apical end points should be conducted in the future. Currently, there is an intention under EU Regulation 1272/2008 on the Classification, Labeling and Packaging of Substances and Mixtures (CLP) to classify TFA and its salts under Category 1B: Presumed human reproductive toxicant, based on new evidence of embryo-fetal developmental toxicity in rabbits, submitted to the European Chemicals Agency (ECHA) by Germany.^52,102,103^ If TFA were to be classified as toxic for reproduction, this could have an impact on future threshold values in food and drinking water. In 2020, Germany established a human-health guideline value for TFA in drinking water at 60 μg/L, which is based on a chronic rat toxicity (feeding) study, but emphasized that the concentration in drinking water should be kept as low as reasonably possible and a value of 10 μg/L should be targeted.^104^ In 2023, The Netherlands derived an indicative drinking water value for TFA at 2.2 μg/L, based on the potency factor relative to perfluorooctanoic acid (PFOA) and its threshold for drinking water.^105^ Considering that the mean and maximum TFA concentrations in German drinking water sources were recently measured at 0.9 and 12.4 μg/L,^2^ respectively, it is plausible that drinking water concentrations in many areas have or will exceed the 2.2 μg/L threshold in the near future.

Under the EU’s CLP Regulation, TFA is classified as harmful to aquatic life with long-lasting effects (H412).^106^ Under REACH, the toxicological threshold for the general population (Derived No Effect Level, DNEL) is derived only for the oral route and set to 0.042 mg/kg_bw_/day, while for other routes of exposure, no hazard has been assumed and concluded. The threshold for the oral route was derived based on the observed effects from a 90-day rat-feeding study.^52^ Human toxicokinetic evaluation of TFA showed rapid oral absorption, submission to enterohepatic circulation, and body distribution via blood, which included passing through the placenta barrier. The main excretion routes are considered to be via urine and bile.^52^ Although TFA is not bioaccumulative according to regulatory criteria referring to aquatic organisms,^52^ internal concentrations may increase due to continuous accumulation in exposure media, such as drinking water and plant-based food and beverages.

Contrary to the most recent human toxicological review,^17^ a similar recent review is not available for (eco)toxicity data. We conducted a search of the USEPA ECOTOX database^107^ and ECHA registration dossiers^52^ and found that most efforts toward the determination of (eco)toxicity of TFA are from the 1990s. Algae are considered the most sensitive trophic level, with *S. capricornutum* the most sensitive species and 120 μg/L the lowest determined No-Observed Effect Concentration (NOEC), which was determined for the TFA sodium salt (Solvay data, ECOTOX database extracted for TFA/NaTFA and reported in Berends et al.^8,107^). Selected ecotoxicity end points (acute and chronic) for other organisms are reported in Table S2 of the Supporting Information, with listed ecotoxicological thresholds and their abbreviations. Overall, most of the testing was performed for acute toxicity with only a few studies investigating the chronic toxicity of TFA.

Predicted no-effect concentrations (PNECs) are concentration thresholds commonly derived in environmental risk assessment that should be protective of the whole ecosystem. They are based on ecotoxicity data and extrapolation factors based on data abundance, test duration (acute/chronic), and data quality/uncertainty.^108^ For TFA, the lowest PNEC of 0.12 μg/L was derived by Xie et al.^83^ and was based on algae that were previously shown as the most sensitive trophic level. The PNEC of 0.12 μg/L was derived from a NOEC of 120 μg/L by using an uncertainty factor of 1000, chosen because of the scarcity of (eco)toxicity data in the peer-reviewed literature and because extrapolation to the marine environment requires extra precaution (typically by a factor of 10 compared to freshwater). The ECHA REACH dossier reports a freshwater PNEC of 560 μg/L and a marine water PNEC of 56 μg/L, both based on the algal study reporting the 72 h ErC10 (EC10 based on growth rate) of 5600 μg/L.^52^ Almost all surface water median and/or mean concentrations from peer-reviewed studies reported as post-2010 already exceed the precautionary PNEC of 0.12 μg/L as derived by Xie et al.^83^ (Table S1, Figure S2), while the NOEC of 120 μg/L and ECHA’s PNEC of 560 μg/L are exceeded by some recently detected maximum freshwater/surface water values.^84,85^

Among terrestrial organisms (ecotoxicity studies), only crop plants were tested for TFA toxicity in the REACH dossier. The EC50 was 4.7 mg/kg soil dry weight, and a long-term NOEC was 0.83 mg/kg soil dry weight. It was mostly the plant shoot growth that was affected.^52^ Further testing of TFA plant toxicity is needed, due to the scarcity of plant toxicity studies with sufficiently high internal plant concentrations, and for including other vascular plants than crops.^13^ However, the long-term NOEC determined in crops^52^ is already similar to soil TFA background concentrations and is several orders of magnitude lower than TFA soil concentrations in contamination hotspots (Figure 1, Figure S4).^5,39^

Given the persistence of TFA, exposure to TFA should be considered chronic and lifelong for all species. However, chronic studies are still relatively scarce, with chronic data from standardized tests being limited in time of exposure to, for example, 35 days in fish, 21 days in *Daphnia*, 90 days in rats, or 36 days in crop plants^52^ (Table S2, SI), which are insufficient in their extrapolation to potential impacts from lifetime exposure to TFA. Hence, none of the current studies considered actual long-term exposure to TFA, which would be more relevant given its ubiquitous and increasing presence over long time scales.

In contrast to the analysis above, other authors^109,110^ recently concluded the risk of TFA to humans and the environment is *de minimis* (i.e., negligible) based on examples like a projected concentration of TFA in the ocean of 0.2 μg/L by 2100 compared to the NOEC in the algae *R. subcapitata* of 2500 μg/L,^109,110^ as well as the median human serum levels of 8.46 μg/L in the serum of staff of Nankai University being 4 orders of magnitude below effect doses in rats. However, we disagree that these are sufficient data to conclude negligible risk, considering that the conclusion ignores terrestrial environments, assumes that the lowest measured environmental effect levels are known despite the lack of long-term chronic exposure data for other species, and does not consider diverse human exposure pathways in the context of increasing TFA concentration in diet and potential unknown adverse effects, such as on embryo development. Given the persistence and accumulation of TFA, there could be other, unknown environmental and health impacts for which the current science is ignorant.

### Unknown Environmental and Health Impacts

As TFA is accumulating in diverse ecosystems, researchers should focus on nontraditional exposure and impact pathways that affect biogeochemical processes. Despite the extremely high plant uptake of TFA, controlled uptake experiments have rarely been performed and reported for TFA. Zhang et al.^56^ performed a set of hydroponic experiments with TFA and other ultrashort and short PFCAs. Here, the root uptake was exceptionally high for TFA when compared to other PFCAs, e.g., the root concentration factor of TFA was >1600 L/kg,^56^ 2 orders of magnitude higher than usually determined for PFCAs with 3–5 perfluorinated carbons in similar experiments.^111−113^ Chen et al.^5^ found a similar pattern for chain length dependence across PFCAs and the field-based soil-water distribution coefficients (*K*_d_). They determined *K*_d_ values that were higher than expected for TFA (and PFPrA), demonstrating the opposite to the commonly observed trend of *K*_d_ decreasing as the perfluoroalkyl chain length decreases from 11 to 3 perfluorinated carbons for PFCAs and PFSAs.^114−117^ This indicates that the interface partitioning of TFA and interactions with soils and plants need to be further researched and accounted for in assessing its environmental fate and behavior properties, particularly its (bio)accumulation potential. Such research needs to be extended to agricultural systems, such as the transfer from crops containing elevated levels of TFA to livestock (meat and milk) and human diets, as well as a transfer of TFA from plants through diverse food webs, similar to previous research on other PFAS.^58−61^

Direct effects of TFA on soil quality were only recently investigated.^118^ Effects on the soil pH, microbial respiration, bacterial abundance and litter decomposition were reported, the latter being affected at concentrations similar to current TFA concentrations in soil for contamination hotspots as described in Chen et al.^5^ (0.0013–2.4 mg/kg_dw_). Effects other than litter decomposition were statistically significant only at high TFA concentrations (≥10 mg/kg) which could be a result of acidity-related effects, as the pH significantly decreased at concentrations of 10 mg/kg and higher (TFA in the acid form was directly used in the testing).^118^ Bott and Standley observed the incorporation of TFA into cells by microbial communities in freshwater surface sediments^119^ after also demonstrating TFA incorporation in biomolecules such as proteins in aquatic organisms spanning a range of trophic levels.^11^ Concentrations that were used in these ∼1.5 to 2.5-year experiments overlap with those currently observed in waters worldwide, ranging from 2.2 to 43 μg/L (Table S1, Figure S2, SI), and resulted in significant cell incorporation of TFA.^119^ It was also indicated that at elevated concentrations, the presence of TFA in the atmosphere may influence aerosols and cloud formation, boosting the formation of atmospheric clusters involved in new aerosol particle formation.^120,121^ As concentrations of TFA will likely increase by at least an order of magnitude in the coming decade, further investigations of TFA on biogeochemical processes are warranted.

## Environmental Implications

Based on the evidence presented above, the increasing accumulation of TFA can be considered to meet the three conditions of a planetary boundary threat^122,123^ for novel entities as defined by Persson et al.:^25^ “*Condition 1 (C1) – the pollution has a disruptive effect on a vital earth system process of which we are ignorant; Condition 2 (C2) – the disruptive effect is not discovered until the associated impacts are, or inevitably will be, manifested at a global scale; and Condition 3 (C3) the impacts are poorly reversible because the level of pollution in the global environment cannot be readily reduced*...” Condition 1 (C1) is fulfilled based on the many thresholds that could be exceeded of which we are ignorant; including surface water concentrations^84,85^ that exceed^83^ ECHA’s PNEC of 560 μg/L; soil concentrations that exceed the lowest established NOEC (0.83 mg/kg soil);^52^ and, indications of human toxicity that have led to precautionary thresholds in drinking water.^104^ The existence of such health advisory and regulatory values has been used as the basis for establishing C1 in relation to four PFAAs in a previous study.^27^ The possibility of other long-term disruptive effects, which may be already occurring but of which we are ignorant, further supports the C1 being fulfilled, particularly considering the absence of studies of TFA exposure in agricultural systems and diverse food webs. Condition 2 (C2) is fulfilled as TFA is already present globally in all environmental media, such as its ongoing bioaccumulation in vascular plants, the accumulation in arctic regions, its ubiquity in groundwater, and the global occurrence of industrial sites that are TFA hotspots where impacts are most likely; therefore, when impacts are discovered, they would be global. Since effects are realized globally and will be irreversible for the foreseeable future, condition 3 (C3) is fulfilled due to TFA’s extreme persistence and mobility coupled with emissions from multiple sources.

Although, currently, TFA does not have as well-established health advisories or regulatory limits as other PFAA,^27^ it is likely that new advisories/limits will be introduced in the coming years when more research on the impacts of TFA emerges. With the projected exponential increase in TFA concentrations in the environment,^81^ food and in humans, the question is not if TFA can exceed a planetary boundary, but which irreversible health or Earth system impacts would be first observed at a planetary scale, and where thresholds of TFA emissions should be set to limit the severity of such impacts.

The potential long-term, irreversible impacts from the rapidly increasing emissions of TFA from anthropogenic sources should be used as a rationale to start immediately discussing policy, industry, and innovation actions toward the phase-out of high-volume substances that lead to increasing TFA accumulation. Obvious places to start would be to limit the direct production of TFA, and more importantly, of TFA-precursors, such as HFOs (like HFO-1234yf) and pesticides such as flufenacet.^13,53,96^ Other pharmaceuticals, veterinary products and industrial chemicals that release TFA via transformation processes^7^ should also be considered for phase-out or substitutions, following the principles of safe-and-sustainable by design^124^ and essential use^125^ to avoid regrettable substitution^126^ to more hazardous substances. As discussions and policy mechanisms to phase out the sources of TFA could take some time, the rational response to the global threat posed by accumulating TFA is to act swiftly before irreversible impacts are manifested at a global scale to humans and the environment. Transitioning away from TFA and its precursors is the most effective way of safeguarding future generations from this planetary boundary threat.

## Supporting Information Available

The monitoring data collection and methodology (PDF)
A full set of collected monitoring data with individual graphics and an overview of selected ecotoxicity values referred to in this study(XLSX)
