# Supplementary material for: The Global Threat from the Irreversible Accumulation of Trifluoroacetic Acid (TFA)
Source: Environ Sci Technol. 2024 Oct 30;58(45):19925–35. doi: 10.1021/acs.est.4c06189 (PMC11562725; doi:10.1021/acs.est.4c06189)
Supplement: Supplementary file 1 — es4c06189_si_001.pdf [file es4c06189_si_001.pdf]

Supplementary Information

for

The global threat from the irreversible accumulation of trifluoroacetic acid (TFA)

by

Hans Peter H. Arp[1,2*], Andrea Gredelj[1], Juliane Glüge[3], Martin Scheringer[3,4]

and Ian T. Cousins[5]

1. Norwegian Geotechnical Institute (NGI), 0484, Oslo, Norway
2. Department of Chemistry, Norwegian University of Science and Technology (NTNU), 7491, Trondheim, Norway
3. Institute of Biogeochemistry and Pollutant Dynamics, ETH Zürich, 8092 Zürich, Switzerland
4. RECETOX, Masaryk University, 625 00 Brno, Czech Republic
5. Department of Environmental Science, Stockholm University, SE-10691 Stockholm, Sweden

Corresponding authors contact: hans.peter.arp@ngi.no

This document contains 6 pages and 4 figures and refers to 2 tables available as an accompanying .xlsx file (Table S1 and Table S2).

Text S1. Monitoring data collection and analysis

To produce an overview of TFA concentrations in environmental matrices and other media, a review of scientific literature was performed via Google Scholar. The intention was not to perform a comprehensive overview of all available data, but rather concentrate on relevant keywords, i.e., “TFA” or “trifluoroacetic acid” AND “environmental concentrations”. In total, 43 studies (including one report on TFA in plant-based foods, [1]) were found to be reporting on monitoring of TFA and/or targeted sampling related to its point or diffuse, direct or indirect sources.

All monitoring/sampling studies were analysed for reported concentrations in different environmental media as minimum, maximum, mean and/or median values. Depending on the reporting and supplementary (raw) data available, mean and/or median concentrations were calculated based on the needs of this perspective (e.g. if authors reported individual samples for multiple rivers of the area, min/max/mean were reported/calculated for all the rivers, regardless of how the authors decided to showcase and interpret their data). All units were converted to ng/L for all water samples and human serum, ng/g (dry weight) for all soil, sediment, dust, sludge, plant and animal samples, pg/m^3 for air. Air samples were reported as total air (gas plus particle) and when reported separately, concentrations were summed, as demonstrated by [2,3].

Apart from the summary of monitoring data reported in the main text as Figure 1, here, a full graphical presentation of data points per study and per environmental media are shown in Figures S1-S4, comparing the periods before and after 2010. Vertical lines are introduced to show the main intervals where a majority of data points are falling into. All data points with corresponding references are reported in Table S1 in the .xlsx supplement.

Precipitation, pre-2010

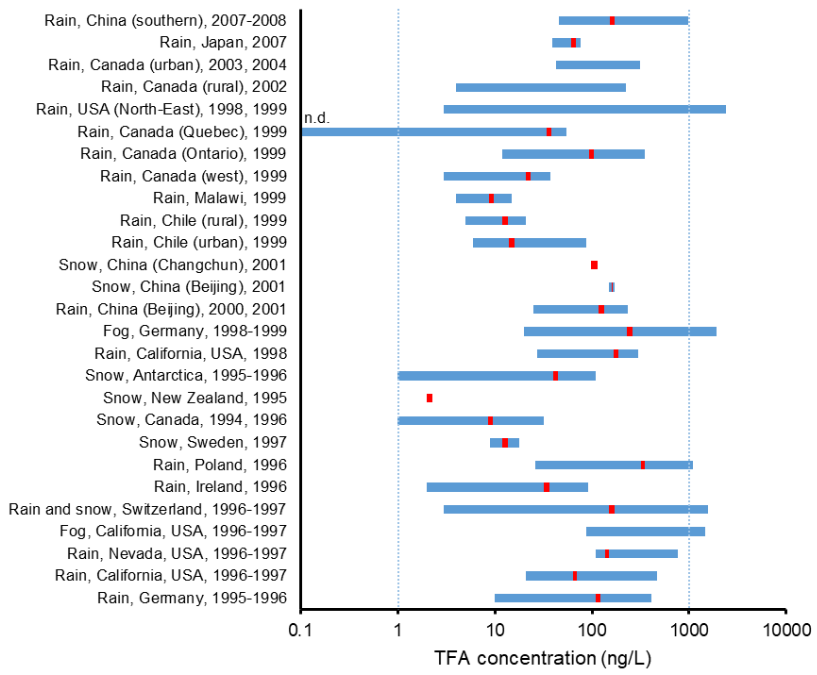

Other water, pre-2010

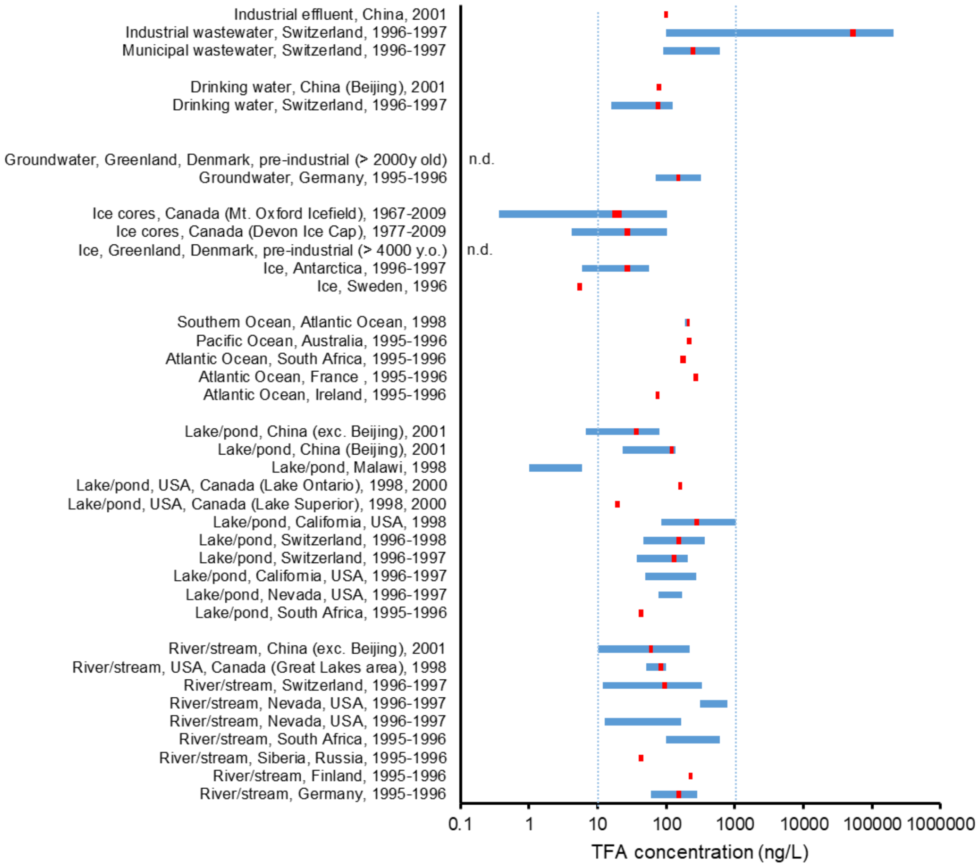

Figure S1. Precipitation and other water samples with TFA concentrations reported per study and per environmental media. Expressed as a minimum to maximum values (blue bars) and mean or median (red marking), sampling before 2010. When minimum values were reported as non-detects, the indication "n.d." is given on the graph. All data used for creating the graphics with full references are reported in Table S1 in the .xlsx supplement in the same chronological order.

All water, post-2010

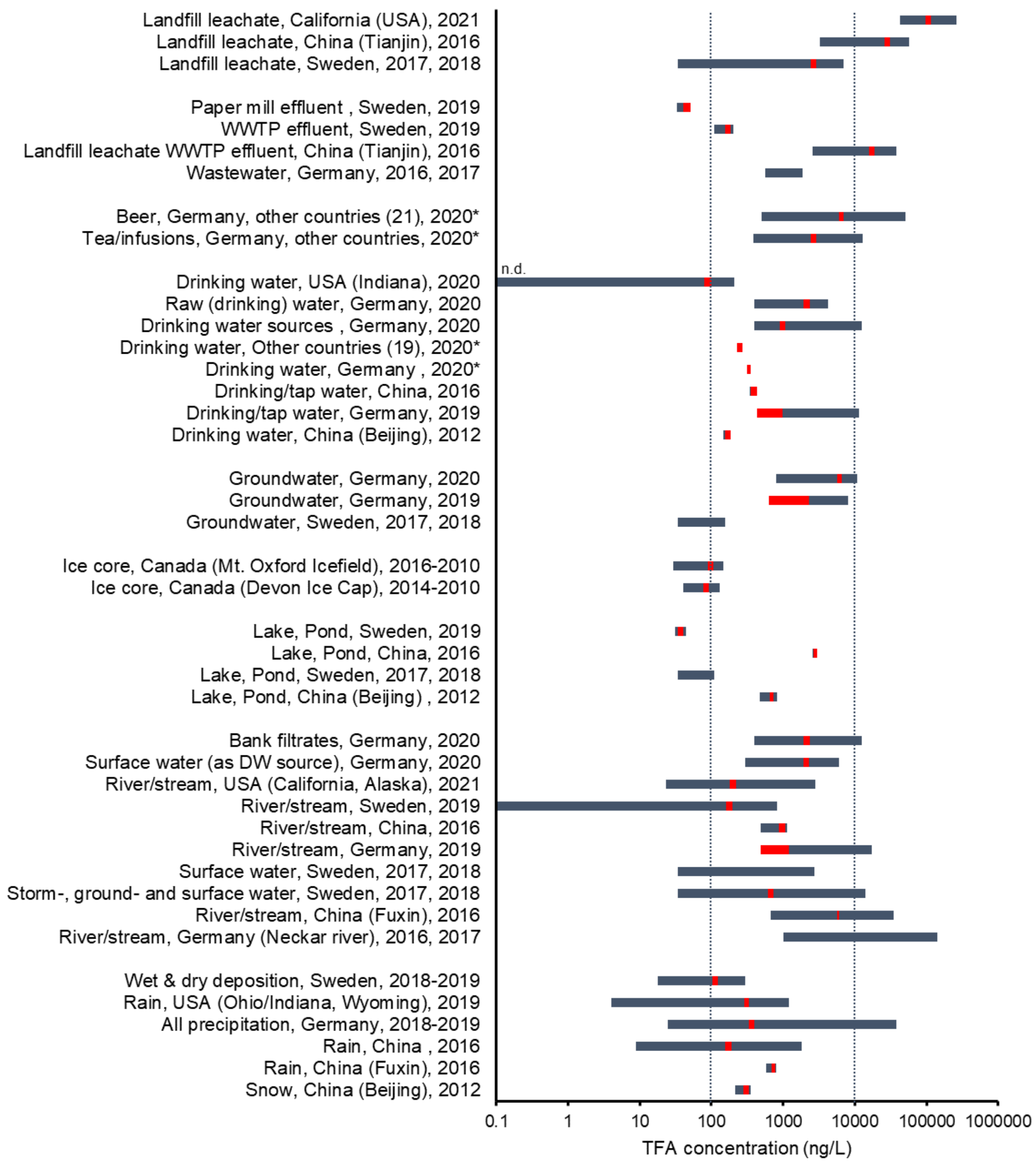

Figure S2. All water samples with TFA concentrations reported per study and per environmental media. Expressed as minimum to maximum values (dark blue bars) and mean or median (red marking), sampling after 2010. When minimum values were reported as non-detects, the indication "n.d." is given on the graph. All data used for creating the graphics with full references are reported in Table S1 in the .xlsx supplement in the same chronological order.

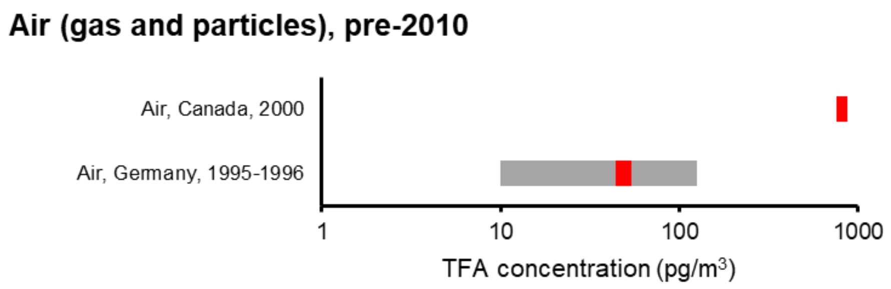

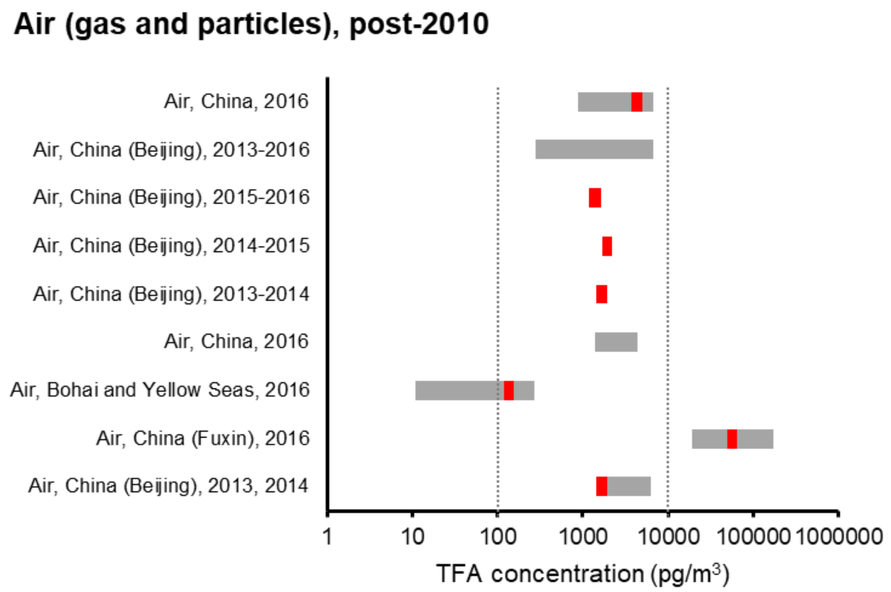

Figure S3. Air samples with TFA concentrations reported per study. Expressed as minimum to maximum values (grey bars) and/or mean or median (red marking) concentrations, the upper graph shows studies with sampling performed before 2010 and the bottom with sampling after 2010. All data used for creating the graphics with full references are reported in Table S1 in the .xlsx supplement in the same chronological order.

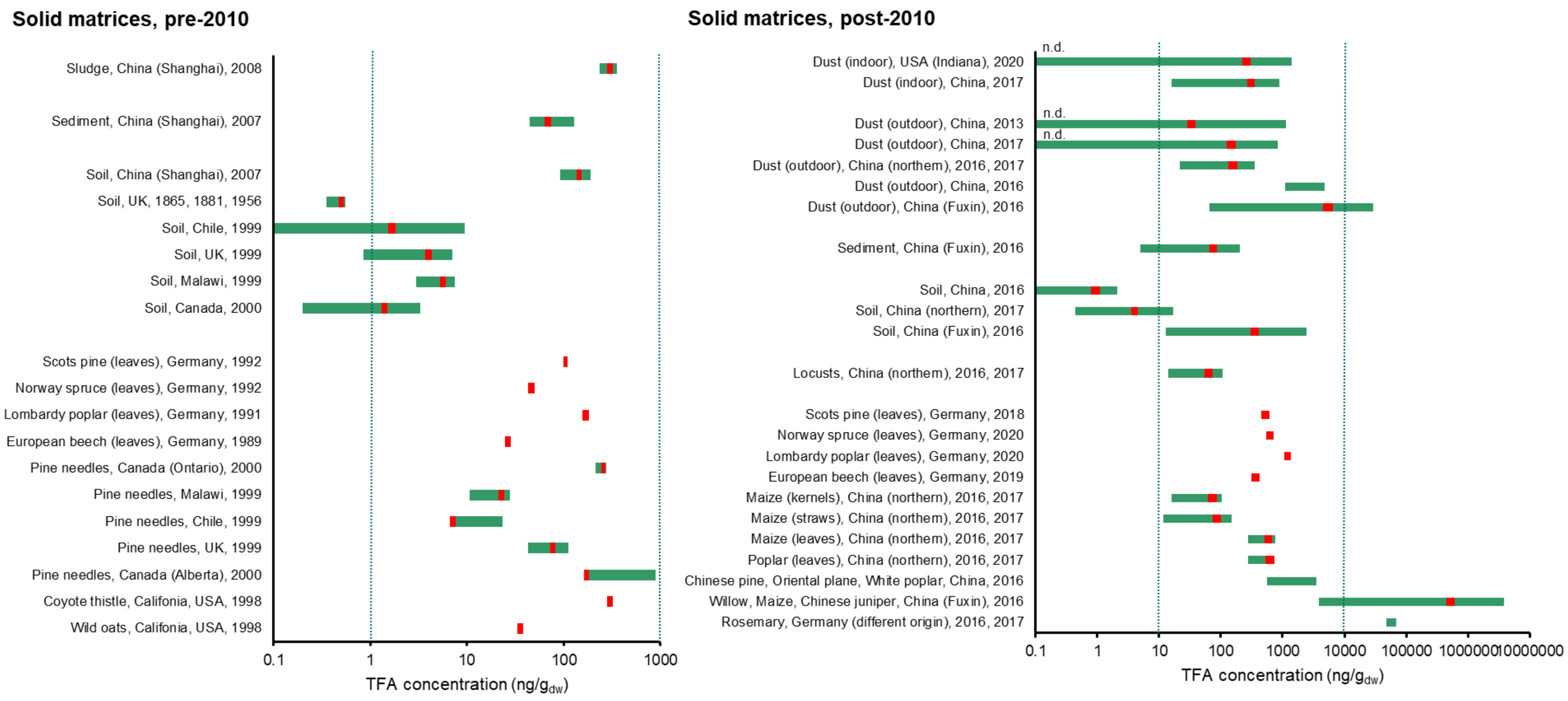

Figure S4. TFA concentrations in studies with different solid matrices, reported per study and per environmental media. Studies reported from sampling before 2010 are shown on the graph on the left-hand side, with sewage sludge, sediment, soil and plant matrices reported. Studies from sampling reported after 2010 are shown on the right-hand side with indoor and outdoor dust, sediment, soil, animals and plants represented. Expressed as minimum to maximum values (green bars) and/or mean or median (red marking) concentrations. When minimum values were reported as non-detects, the indication "n.d." is given on the graph. All data used for creating the graphics with full references are reported in Table S1 in the .xlsx supplement in the same chronological order.

References:

(1) EU Reference Laboratories for Residues of Pesticides. EURL-SRM-Residue Findings Report; 2017. https://www.eurl-pesticides.eu/userfiles/file/eurlsrm/eurlsrm_residue-observation_tfa-dfa.pdf (accessed 2023-12-08).

(2) Zhang, B.; Zhai, Z.; Zhang, J. Distribution of Trifluoroacetic Acid in Gas and Particulate Phases in Beijing from 2013 to 2016. Science of the Total Environment 2018, 634, 471–477. https://doi.org/10.1016/j.scitotenv.2018.03.384.

(3) Fang, X.; Wang, Q.; Zhao, Z.; Tang, J.; Tian, C.; Yao, Y.; Yu, J.; Sun, H. Distribution and Dry Deposition of Alternative and Legacy Perfluoroalkyl and Polyfluoroalkyl Substances in the Air above the Bohai and Yellow Seas, China. Atmos Environ 2018, 192, 128–135. https://doi.org/10.1016/j.atmosenv.2018.08.052.
